# Maternal Supplementation with Antioxidant Vitamins in Sheep Results in Increased Transfer to the Fetus and Improvement of Fetal Antioxidant Status and Development

**DOI:** 10.3390/antiox8030059

**Published:** 2019-03-08

**Authors:** Francisco Sales, Oscar A. Peralta, Eileen Narbona, Sue McCoard, Raúl Lira, Mónica De Los Reyes, Antonio González-Bulnes, Víctor H. Parraguez

**Affiliations:** 1 INIA-Kampenaike, Punta Arenas 6212707, Chile; fsales@inia.cl (F.S.); rlira@inia.cl (R.L.); 2 Faculty of Veterinary Sciences, University of Chile, Santiago 8820808, Chile; operalta@uchile.cl (O.A.P.); eileen.narbona@gmail.com (E.N.); mdlreyes@uchile.cl (M.D.L.R.); 3 AgResearch Grasslands, Palmerston North 4442, New Zealand; sue.mccoard@agresearch.co.nz; 4 INIA-Madrid, Ciudad Universitaria s/n, 28040 Madrid, Spain; bulnes@inia.es; 5 Facultad de Veterinaria, Universidad Complutense de Madrid, Ciudad Universitaria s/n, 28040 Madrid, Spain; 6 Faculty of Agricultural Sciences, University of Chile, Santiago 8820808, Chile

**Keywords:** maternal-fetal vitamins transfer, fetal antioxidant capacity, fetal growth

## Abstract

Twinning and maternal nutritional restriction leads to fetal hypoxia, oxidative stress, and intrauterine growth restriction (IUGR) in near-term sheep pregnancies. Our aim was to determine the effect of oral supplementation of vitamins C and E in pregnant sheep on maternal and umbilical cord blood concentrations of vitamins C and E and the effects on fetal antioxidant status, growth, and placental efficiency. Sixteen single- and sixteen twin-bearing ewes, grazing natural Patagonian prairies, were selected after transrectal ultrasound at day 30 after mating. Half of ewes from each pregnancy rank were supplemented daily with vitamins C and E, administered orally, from 30 to 140 days of gestation, when maternal jugular and fetal venous cord blood samples were obtained during cesarean section. Fetuses were weighed and sexed. Placental weight in each fetus was also obtained. Blood plasma was harvested for measurements of maternal and fetal vitamins concentration and fetal antioxidant capacity. Maternal administration of vitamin C and E was associated with increased fetal cord levels of both vitamins, improved antioxidant status, and enhanced fetal growth in both singleton and twin pregnancies associated with increased placental efficiency. These results highlight the potential of vitamin C and E supplementation to reduce the impact of IUGR in both livestock and humans.

## 1. Introduction

Intrauterine growth restriction (IUGR) is the failure of fetuses to reach their genetically established growth rate resulting in low-birth-weight (LBW) offspring and is a concerning issue for both human and veterinary medicine. IUGR has profound implications for mortality and morbidity of LBW neonates and has long-term consequences for growth, health, and performance of these individuals.

Occurrence of IUGR is mainly related to inadequate supply of nutrients and oxygen to the fetus, due to maternal malnutrition and/or placental insufficiency [1,2,3]. The sheep has been extensively used as a model for studies on IUGR due to its usefulness for both animal production and biomedical studies [4] and early studies have highlighted the effect of nutrition on fetal growth patterns in sheep [5]. A key feature of sheep, like in humans, is the occurrence of singleton and twin pregnancies. In twinning, maternal undernutrition has a greater impact on fetal growth [6] as twin pregnancies are often related to IUGR induced by placental insufficiency [7,8,9]. Twinning, with or without concurrent maternal undernutrition, negatively affects placental development through reduced individual number of placentomes and total placental mass [10]. Such deficiencies in placental development cause inadequate nutrient supply and reduced oxygenation of the fetus [11,12]. Hypoxia increases oxidative stress and aggravates IUGR effects, as found in fetuses exposed to maternal hypobaric hypoxia [13]. Recently we have also demonstrated that twining and maternal nutritional restriction leads to fetal hypoxia and oxidative stress in near-term sheep pregnancies [14].

The occurrence and extent of IUGR being primarily related to a weakened antioxidant defense system [15,16] may be alleviated by the improvement of oxidative stress status during pregnancy [17,18,19]. Hence, the supplementation with antioxidant agents may be beneficial for IUGR-compromised pregnancies, as found in previous studies in sheep, in which the deleterious effects of maternal hypoxia-induced oxidative stress may be prevented by the administration of antioxidant vitamins [13,20]. However, to the best of our knowledge, there is a scarcity of information on the effectiveness of maternal supplementation with antioxidant vitamins during pregnancy in sheep, and notably, their transfer across the placenta and thus fetal availability, and effects on fetal antioxidant status and growth.

Therefore, the purpose of this study was to determine the concentrations of vitamins C and E in maternal and umbilical cord blood after oral supplementation of pregnant sheep, and the effects of such supplementation on the antioxidant status and growth of the fetus. Potential differential effects due to pregnancy rank (singleton or twins) were also assessed. These results have direct implications for the proposed practice of supplementing pregnant sheep with vitamins C and E for preventing or counteracting IUGR, but may also be of translational value to other species and even to human biomedical research.

## 2. Materials and Methods

### 2.1. Animals and Experimental Procedure

The present study was carried out at the INIA (National Institute for Agricultural Research) research farm, 65 km north from Punta Arenas, in the Magellan Region (Chilean Patagonia; latitude 52°36′, longitude 70°56′), according to the Guide for Care and Use of Laboratory Animals (Eighth Edition, National Research Council, National Institute of Health, Washington, DC, USA). The experimental procedure was approved by the Bioethics Committee of the INIA and the Bioethics Review Committee of the Faculty of Veterinary Sciences, University of Chile (Protocol # 11-2016), as well as by the Bioethics Advisory Committee of the Chilean National Commission for Scientific and Technological Research (CONICYT, Chile) as funder of the study.

The trial involved a total of 32 Corriedale ewes, 3–5 years old, 62.3 ± 1.2 kg body-weight and 2.0–2.5 body-condition-score (BCS; scale 0–5) [21], carrying singleton (*n* = 16) or twin pregnancies (*n* = 16). These females were randomly selected from a group of 200 ewes belonging to the INIA experimental flock. Estrous cycles were synchronized with intravaginal controlled internal drug release (CIDR) devices (CIDR G^®^, Pfizer, Chile) and maintained for 12 days, followed by a single i.m. dose of 300 IU equine Chorionic Gonadotrophin (eCG, Novormon^®^, Syntex, Argentine) at CIDR removal, with the purpose of obtaining the necessary amount of twin pregnancies for the study. Mating was carried out using 20 fertile proved Suffolk rams with painted chests using a solution of colored earth in food-grade oil, for detection of the exact day of service by daily visual inspection of ewe’s colored rumps.

The experimental model was a 2 × 2 factorial design with two treatments (supplemented and non-supplemented with vitamins) and two pregnancy ranks (singleton and twins). Hence, four experimental groups were formed (*n* = 8 ewes in each group): Two groups of singleton pregnancies, with or without vitamin supplementation (groups SC and SV, respectively), and two groups of twin pregnancies, with or without vitamin supplementation (groups TC and TV, respectively). The number of animals per group was calculated according to variability of fetal total antioxidant capacity at 140 days of gestation previously observed [14], considering a statistical power of 95% and α = 0.05. These groups were established after transrectal ultrasound pregnancy diagnosis at day 30 of pregnancy (~20% of the total length of ovine pregnancy, estimated in a mean of 148 days). The groups treated with vitamins (groups V) supplemented daily with 500 mg of vitamin C (ascorbic acid) and 350 IU of vitamin E (α-tocopherol) (Veterquímica, Chile) *per os*, from day 30 of pregnancy onwards. The selected doses of vitamins C and E have been shown to significantly increase the maternal plasma concentrations of both vitamins in pregnant sheep [13]. All the ewes were managed under commercial field conditions, grazing natural Patagonian pastures, which is insufficient to fully satisfy the nutritional requirements of pregnancy [14].

The evaluation of maternal plasma vitamin levels following supplementation and the transfer of the vitamins to the fetus(es) and potential changes in their antioxidant status was performed near term (140 days of gestation; ~95% of the total length of ovine pregnancy). Heparinized (Sodic Heparine^®^, Sanderson, Chile) blood samples were collected from the mother by jugular venipuncture, whilst fetal cord samples were obtained by umbilical venipuncture, during cesarean section, performed under maternal spinal anesthesia by injection of 2 mL 2% Lidocaine hydrochloride (Lidocalm^®^, Drag Pharma, Chile) into the sacrococcygeal space. Immediately after collection, blood was centrifuged at 1200× *g* for 10 min to obtain the plasma which was separated and stored into polypropylene vials at −80 °C until assayed. The fetus(es) and ewe were immediately euthanized by barbiturate overdose (Opet^®^, Pro-Vet, Chile), and fetuses and placentas were extracted. Excess of fetal fluids were hand stripped and the fetal weight and sex were recorded. Total placentome weight was obtained after dissecting and weighing each placentome individually. Furthermore, placental efficiency was also calculated in accordance to Richter et al. [22].

Measurement of the concentrations of vitamin C and E in plasma was performed by high-performance liquid chromatography as previously described [13].

Assessment of the total antioxidant capacity (TAC) in plasma was performed using a colorimetric antioxidant assay kit (Cayman Chemical Company, Ann Arbor, MI, USA), according to the instructions of the manufacturer and our own experience with ovine blood plasma [23].

### 2.2. Statistical Analysis

Analysis of variance (GLM; SAS Institute Inc., Cary, NC, USA) was used for determining the effects of treatment (control vs. vitamins supplementation), pregnancy rank (single and twin) and their interactions. The model did not include the effect of fetal sex, since this was found not statistically significant in a first test. The Pearson correlations between maternal plasma vitamins concentrations and fetal cord vitamin concentrations, as well as between these last and fetal body weight and fetal TAC, were calculated. In addition, a multiple correlation between fetal plasma vitamins concentrations and fetal TAC was also calculated. The differences were considered significant when *p* ≤ 0.05. The results were expressed as means ± SEM.

## 3. Results

**Figure 1:**
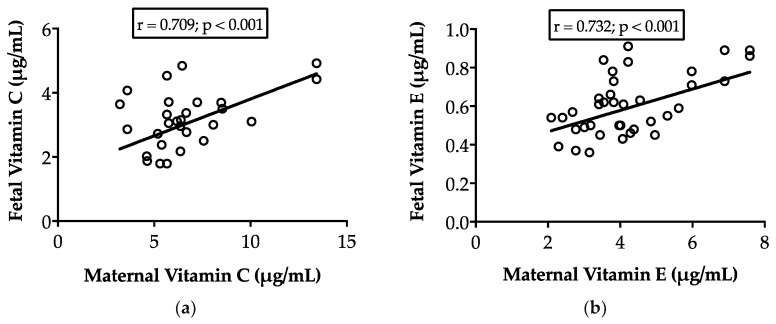
Correlations between maternal and fetal cord blood concentrations of vitamins C (**a**) and E (**b**) at 140 days of pregnancy, in ewes supplemented daily with vitamins C (500 mg) and E (350 IU) from day 30 after mating.

Oral maternal supplementation with vitamins C and E increased maternal plasma concentrations of both vitamins (*p* < 0.05), irrespective of pregnancy rank (Table 1). Our data also showed that both vitamins were directly transferred to the fetuses since there was a strong positive correlation, in both non-supplemented control and supplemented fetuses, between maternal and fetal concentrations of vitamin C (*r* = 0.709; *p* < 0.001) and vitamin E (*r* = 0.732; *p* < 0.001), as depicted in Figure 1. Hence, maternal supplementation with vitamins C and E also increased the concentrations of both vitamins in the samples obtained from fetal cord blood. Consequently, treated fetuses showed higher concentrations of vitamin C (3.58 ± 1.17 µg/mL vs. 2.84 ± 0.72 µg/mL in the control group; *p* < 0.01) and vitamin E (0.66 ± 0.15 µg/mL vs. 0.55 ± 0.13 µg/mL in the control group; *p* < 0.05) (Table 1). 

The TAC in fetal cord blood was higher in vitamin-supplemented pregnancies (*p* < 0.01; Table 1). No significant correlations were observed between fetal TAC and maternal or fetal concentrations of vitamins C and E (*p* > 0.05). However, multivariate analysis showed that fetal TAC was positively related when considered simultaneously the effects of vitamin C and E concentrations at fetal cord blood (*r* = 0.883, *p* < 0.05).

Pregnancy rank affected vitamin concentrations and TAC in fetal cord blood, independently of vitamin supplementation, since no interaction between vitamin supplementation and pregnancy rank was found. Singleton fetuses had significantly lower concentrations of both vitamin C and E (*p* < 0.001) and a trend for higher TAC than twin fetuses (*p* = 0.06) in both control and supplemented pregnancies (Table 1).

The assessment of fetal weight showed that singleton fetuses were heavier than twins (*p* < 0.01) in both non-supplemented and supplemented pregnancies. Maternal supplementation with vitamins C and E increased fetal weight (*p* < 0.05) irrespective of pregnancy rank, however, a greater effect was observed in twins compared to singletons (15% vs. 8%, respectively). No vitamin supplementation by pregnancy rank interaction was found (Table 1). The increase in fetal weight was positively associated with maternal and fetal concentrations of vitamin C (*r* = 0.428 and *r* = 0.424, respectively; *p* < 0.05), vitamin E (*r* = 0.605 and *r* = 0.547, respectively; *p* < 0.0005) and fetal TAC (*r* = 0.308, *p* = 0.05). 

Total placentome weight per fetus was higher in singletons compared to twins (*p* < 0.001). No effect of the vitamins supplementation for this trait and no interaction between pregnancy rank and vitamins supplementation were observed (*p* > 0.05 in both cases; Table 1). Placental efficiency, however, was greater in both twins and vitamin supplemented pregnancies compared to singletons and unsupplemented controls respectively (*p* = 0.007 for both effects). As for the other traits, no interaction between pregnancy rank and vitamins supplementation was observed (*p* > 0.05).

## 4. Discussion

To our knowledge, this is the first study demonstrating that oral supplementation with vitamin C and vitamin E to single- and twin-bearing ewes from day 30 of gestation, results in a significant increase in the concentration of both vitamins in fetal cord plasma. The increase of circulating concentration of both vitamins in maternal plasma as a consequence of maternal oral supplementation has been previously shown [13]. However, the improvement in the fetal antioxidant status, in addition to the increase of their body-weight independently of pregnancy rank, under normal farming management conditions at sea level, and resulting from increased fetal plasma vitamin concentration, is a novel finding. The present study highlights the potential of strategic interventions with oral antioxidant vitamins during pregnancy, in order to ameliorate the negative impact resulting from maternal stressors, such as twin pregnancy.

In the present trial, maternal oral supplementation with vitamins C and E during almost all of the pregnancies lead to significant increases of both vitamins in ewe blood plasma. This result is consistent with previous studies of our group in pregnant ewes [13] and also in rams after 30 days of treatment [23] using the same oral doses of both vitamins. It is important to highlight that only in recent years it has been accepted that oral vitamin C supplementation is efficient to increase its plasma concentration in ruminants. In a former study, it was reported that vitamin C supplied orally in cows, is destroyed by ruminal microorganisms, and then it is unable to reach the blood circulation and other territories [24]. This was later confirmed in sheep [25]. However, the opposite was demonstrated in other studies showing that long-term oral supplementation with both vitamin C and E in ruminant’s leads to increase these vitamins in the blood, which is described in Parraguez et al. [13].

Fetal body weight was higher in single than in twin pregnancies, which has been systematically reported in sheep and other species [6,26,27,28]. Maternal supplementation with vitamins C and E lead to a 5.8 and 14.5% increase in fetal body weight in singletons and twins, respectively, indicating a greater effect in twins. The fetal weight response to antioxidant vitamins, obtained in twins is consistent with the presence of fetal hypoxia and oxidative stress previously described for this type of pregnancy [14], and with the effects of vitamins to counteract the action of maternal hypoxia and oxidative stress on fetal growth [13]. However, in the last cited study, no effect of maternal vitamins supplementation was seen in pregnancies bearing singletons developed under normoxic conditions in absence of maternal oxidative stress. A probable explanation for the difference between that study and the present results may be due to differential planes of maternal nutrition. The ewes used in the cited study were maintained under controlled conditions, with a feed supply enough to satisfy requirements; in contrast, the ewes in the present trial were maintained under field Patagonian conditions, whereby pastures do not cover the requirements of pregnancy, leading to decrease fetal oxygenation and a slight decrease in total antioxidant capacity [14]. These results suggest antioxidant vitamins would have a greater impact in undernourished maternal regimes.

The total placentome weight per fetus obtained as the sum of individual weights of all placentomes after dissecting and weighing each individual placentome, was higher in single than in twin pregnancies, which is in agreement with previous reports [26,29]. Supplementation with vitamins did not have an effect on placental weight consistent with a previous observation in pregnant sheep managed at sea level [13]. According to our knowledge, in the literature there are no other studies, in addition to those cited earlier concerning the combined supplementation of vitamins C and E on sheep placental weight. However, reports on the effects of supplementation of pregnant sheep with antioxidant compounds like selenium [30] or melatonin [31], show that these antioxidants also do not affect placental weight.

As a result of fetal and placental changes, placental efficiency (defined as gram of fetus produced per gram of placenta) was affected by both pregnancy rank and vitamins supplementation. Twin bearing and vitamins supplemented pregnancies had better placental efficiency indexes. Placental efficiency was about 20% higher in twins than in singleton pregnancies, which is in agreement with a previously reported study, which indicated that placental efficiency increases with litter size [32]. In contrast, Ocak et al. [26] indicated a lesser placental efficiency for twin pregnancies in comparison with single ones. However, the latter study considered the total placental weight of both twins, without differentiation of individual placentas of each fetus, which also contrasts with our study. Placental efficiency was increased by vitamins supplementation, an effect that is discussed in the next paragraphs; however, this seems to be a shared effect between different types of antioxidants [33]. The use of melatonin, a potent antioxidant, in undernourished sheep during pregnancy, improves placental efficiency by upregulating placental antioxidant enzymes [22] and reinforcing the potential role of antioxidants, such as vitamins E and C, to improve placental efficiency and fetal outcome.

The concentration of vitamin C found in fetal cord blood, suggests efficient placental transfer, in agreement with previous studies in guinea pigs [34,35]. The doses of vitamin C used in the present study were able to increase vitamin C concentrations in fetal plasma by 20% in singletons and 37% in twin fetuses. The concentration of vitamin C reached in fetal plasma was within the concentration range required for acting in vivo as an antioxidant [36], which confirms that the dosage of vitamin C given to the sheep was adequate. The mode of action of vitamin C supplementation may be based in the increase of nitric oxide bioavailability [36], which in turn favors placental angiogenesis and neovascularization and increases umbilical blood flow to the fetus to support fetal growth and development [37].

While maternal vitamin E supplementation increased fetal vitamin E concentrations relative to non-supplemented controls, the concentrations in fetal plasma were very low compared to maternal plasma (13–19% of maternal concentrations), contrasting with the 34–62% observed for fetal-to-maternal ratio of vitamin C. Reduced concentration of vitamin E in fetuses compared to maternal plasma concentration is consistent with previous studies in pigs [38] and humans [39,40,41]. These observations support the notion that liposoluble vitamins, like vitamin E, have a much lower placental transfer rate than hydrosoluble vitamins, like vitamin C, as they require facilitated diffusion and therefore, do not achieve the same concentration in the developing fetus [42]. Placental transfer of vitamin E is not completely understood, but there is evidence indicating a pivotal role of α-Tocopherol Transfer Protein (α-TTP), a cytosolic protein primarily described in the liver with high affinity for α-tocopherol, which can be upregulated in case of oxidative stress [43]. Although expression of α–TTP was not measured in the present study, we have previously shown that twin-fetuses exhibit higher levels of oxidative stress markers than singletons [14], which would explain the higher levels of vitamin E found in twin fetuses in our study, where the TAC level is lower than in singletons and therefore, oxidative stress may be augmented.

Despite the low levels of vitamin E found at the fetoplacental unit, the importance of this vitamin in fetal development is unequivocal [44], not only because it promotes placental angiogenesis [45], but also is component of enzymes in critical pathways to enhance immune function [46]. Furthermore, previous research indicates that supplementation with vitamin E enhances the expression and activity of antioxidant enzymes, such as superoxide dismutase and catalase, and that such effect remains for more than two months [47]. Therefore, the increase in fetal growth observed in the present study in both single and twin fetuses in response to maternal supplementation of vitamins C and E is mediated by increased placental efficiency, which could be modulated, at least in part, through increased placental angiogenesis and placental blood flow. Concomitantly, there is increasing evidence that the combination of vitamins C and E together is more effective than separately [48], which includes pregnancy outcomes [44]. The results of the present study are consistent with these prior studies, in which the improved antioxidant capacity of fetuses was related to the combined action of both vitamins.

## 5. Conclusions

The joint maternal administration of vitamin C and E was associated with increased fetal cord levels of both vitamins, improved antioxidant status and enhanced fetal growth in both singleton and twin pregnancies, but with a greater effect in twins. These results highlight the potential of vitamin C and E supplementation to reduce the impact of IUGR. Such result is of paramount importance in twin pregnancies, in which offspring growth and therefore, postnatal survival and development is compromised. Hence, our results open a new line of action for developing future strategies aiming to prevent or counteract the occurrence of IUGR in sheep. These results are, concomitantly, of translational value to human medicine, in which the impact of twinning, and especially discordant twinning, is a main clinical issue.

## Figures and Tables

**Table 1:** Effects of maternal supplementation of vitamins C and E from day 30 after mating on maternal and fetal cord blood vitamin concentrations, fetal cord total antioxidant capacity (TAC), fetal body weight, placentome weight and placental efficiency at 140 days of pregnancy in ewes bearing single and twin fetuses.

	SC	SV	TC	TV	*p*-Value		
					V	R	VxR
Maternal vit. C (µg/mL)	5.21 ± 0.69	8.09 ± 1.73	5.24 ± 0.27	7.60 ± 0.76	0.028	ns	ns
Maternal vit. E (µg/mL)	3.33 ± 0.33	4.40 ± 0.25	3.18 ± 0.16	5.01 ± 0.39	0.001	ns	ns
Fetal Cord vit. C (µg/mL)	2.26 ± 0.17	2.72 ± 0.28	3.26 ± 0.18	4.45 ± 0.19	0.007	<0.001	ns
Fetal Cord vit. E (µg/mL)	0.47 ± 0.03	0.55 ± 0.03	0.61 ± 0.04	0.72 ± 0.04	0.022	<0.001	ns
Fetal Cord TAC (mM Trolox equiv.)	0.43 ± 0.32	1.06 ± 0.16	0.21 ± 0.10	0.64 ± 0.08	0.006	0.060	ns
Fetal body weight (kg)	3.81 ± 0.19	4.03 ± 0.21	2.82 ± 0.08	3.23 ± 0.07	0.023	<0.01	ns
Total placentome weight (g)	473.5 ± 26.5	431.4 ± 31,8	322.8 ± 17.8	307.9 ± 18.8	ns	<0.001	ns
Placental efficiency	7.70 ± 0.56	9.51 ± 0.45	9.53 ± 0.51	11.18 ± 0.51	0.007	0.007	ns

Data are the mean ± SEM. SC: singleton pregnancies without vitamins supplementation (controls); SV: singleton pregnancies supplemented with vitamins C and E; TC: Twin pregnancies without vitamins supplementation (controls); TV: Twin pregnancies supplemented with vitamins C and E; V: Vitamins effect; R: Pregnancy rank effect; VxR: Interaction between vitamin and pregnancy rank effects. ns: Not significant (*p* > 0.05).

## References

[1] Brodsky D., Christou H. (2004). Current concepts in intrauterine growth restriction. J. Intens. Care Med..

[2] Nardozza L.M., Araujo Júnior E., Barbosa M.M., Caetano A.C., Lee D.J., Moron A.F. (2012). Fetal growth restriction: current knowledge to the general Obs/Gyn. Arch. Gynecol. Obstet..

[3] Cetin I., Mando C., Calabrese S. (2013). Maternal predictors of intrauterine growth restriction. Curr. Opin. Clin. Nutr. Metab. Care.

[4] Gonzalez-Bulnes A., Astiz S., Parraguez V.H., Garcia-Contreras C., Vazquez-Gomez M. (2016). Empowering Translational Research in Fetal Growth Restriction: Sheep and Swine. Anim. Mod. Curr. Pharm. Biotechnol..

[5] Charlton V., Johengen M. (1985). Effects of intrauterine nutritional supplementation on fetal growth retardation. Biol. Neonate.

[6] Rumball C.W.H., Harding J.E., Oliver M.H., Bloomfield F.H. (2008). Effects of twin pregnancy and periconceptional undernutrition on maternal metabolism, fetal growth and glucose-insulin axis function in ovine pregnancy. J. Physiol..

[7] Freetly H.C., Leymaster K.A. (2004). Relationship between litter birth weight and litter size in six breeds of sheep. J. Anim. Sci..

[8] Gootwine E., Spencer T.E., Bazer F.W. (2007). Litter-size-dependent intrauterine growth restriction in sheep. Animal.

[9] Gardner D.S., Buttery P.J., Daniel Z., Symonds M.E. (2007). Factors affecting birth weight in sheep: Maternal environment. Reproduction.

[10] van der Linden D.S., Sciascia Q., Sales F., McCoard S.A. (2014). Placental nutrient transport is affected by pregnancy rank in sheep. J. Anim. Sci..

[11] Westgate J.A., Wassink G., Bennet L., Gunn A.J. (2005). Spontaneous hypoxia in multiple pregnancies is associated with early fetal decompensation and enhanced T-wave elevation during brief repeated cord occlusion in near-term fetal sheep. Am. J. Obstet. Gynecol..

[12] Rurak D., Bessette N.W. (2013). Changes in fetal lamb arterial blood gas and acid-base status with advancing gestation. Am. J. Physiol. Regul. Integr. Comp. Physiol..

[13] Parraguez V.H., Atlagich M., Araneda O., García C., Muñoz A., De los Reyes M., Urquieta B. (2011). Effects of antioxidant vitamins on newborn and placental traits in gestations at high altitude: Comparative study in high and low altitude native sheep. Reprod. Fert. Dev..

[14] Sales F., Peralta O.A., Narbona E., McCoard S., De los Reyes M., González-Bulnes A., Parraguez V.H. (2018). Hypoxia and Oxidative Stress Are Associated with Reduced Fetal Growth in Twin and Undernourished Sheep Pregnancies. Animals.

[15] Gupta P., Narang M., Banerjee B.D., Basu S. (2004). Oxidative stress in term small for gestational age neonates born to undernourished mothers: A case control study. BMC Pediatr..

[16] Biri A., Bozkurt N., Turp A., Kavutcu M., Himmetoglu O. (2007). Role of oxidative stress in intrauterine growth restriction. Gynecol. Obstet. Investig..

[17] Burton G.J., Yung H.W., Cindrova-Davies T., Charnock-Jones D.S. (2009). Placental endoplasmic reticulum stress and oxidative stress in the pathophysiology of unexplained intrauterine growth restriction and early onset preeclampsia. Placenta.

[18] Myatt L. (2010). Review: Reactive oxygen and nitrogen species and functional adaptation of the placenta. Placenta.

[19] Jauniaux E., Burton G.J. (2016). The role of oxidative stress in placental-related diseases of pregnancy. J. Gynecol. Obstet. Biol. Reprod. (Paris).

[20] Parraguez V.H., Urquieta B., De los Reyes M., González-Bulnes A., Astiz S., Muñoz A. (2013). Steroidogenesis in sheep pregnancy with intrauterine growth retardation by high-altitude hypoxia: effects of maternal altitudinal status and antioxidant treatment. Reprod. Fert. Dev..

[21] Jefferies B.C. (1961). Body condition scoring and its use in management. Tasm. J. Agric..

[22] Richter H.G., Hansell J.A., Raut S., Giussani D.A. (2009). Melatonin improves placental efficiency and birth weight and increases the placental expression of antioxidant enzymes in undernourished pregnancy. J. Pineal. Res..

[23] Cofré E., Peralta O.A., Raggi A., De Los Reyes M., Sales F., González-Bulnes A., Parraguez V.H. (2017). Ram semen deterioration by short-term exposure to high altitude is prevented by improvement of antioxidant status. Animal.

[24] Knight C.A., Dutcher R.A., Guerrant N.B., Bechtel S. (1941). Destruction of ascorbic acid in the rumen of dairy cows. J. Dairy Sci..

[25] Hidiroglou M., Batra T.R., Zhao X. (1997). Comparison of vitamin C bioavailability after multiple or single oral dosing of diferente formulations in sheep. Reprod. Nutr. Dev..

[26] Ocak S., Emsen E., Köycegiz F., Kutluca M., Önder H. (2009). Comparison of placental traits and their relation to litter size and parity weight in sheep. J. Anim. Sci..

[27] Dhakal K., Maltecca C., Cassady J.P., Baloche G., Williams C.M., Washburn S.P. (2013). Calf birth weight, gestation length, calving ease, and neonatal calf mortality in Holstein, Jersey, and crossbred cows in a pasture system. J. Dairy Sci..

[28] Blickstein I. (2002). Normal and abnormal growth of multiples. Semin. Neonatol..

[29] Meyer K.M., Koch J.M., Jayanth Ramadoss J., Kling P.J., Magness R.R. (2010). Ovine surgical model of uterine space restriction: Interactive effects of uterine anomalies and multifetal gestations on fetal and placental growth. Biol. Reprod..

[30] Lekatz L.A., Caton J.S., Taylor J.B., Reynolds L.P., Redmer D.A., Vonnahme K.A. (2010). Maternal selenium supplementation and timing of nutrient restriction in pregnant sheep: Effects on maternal endocrine status and placental characteristics. J. Anim. Sci..

[31] Lemley C.O., Meyer A., Camacho L.E., Neville T.L., Newman D.J., Caton J.S., Vonnahme K.A. (2012). Melatonin supplementation alters uteroplacental hemodynamics and fetal development in an ovine model of intrauterine growth restriction. Am. J. Physiol. Regul. Integr. Comp. Physiol..

[32] Dwyer C.M., Calvert S.K., Farish M., Donbavand J., Pickup H.E. (2005). Breed, litter and parity effects on placental weight and placentome number, and consequences for the neonatal behaviour of the lamb. Theriogenology.

[33] Lemley C.O., Vonnahme K.A. (2017). Physiology and endocrinology symposium: Alterations in uteroplacental hemodynamics during melatonin supplementation in sheep and cattle. J. Anim. Sci..

[34] Norkus E.P., Bassi J., Rosso P. (1979). Maternal-fetal transfer of ascorbic acid in the guinea pig. J. Nutr..

[35] Das S., Powers H.J. (1998). The effects of maternal intake and gestational age on materno-fetal transport of vitamin C in the guinea-pig. Br. J. Nutr..

[36] Thakor A.S., Richter H.G., Kane A.D., Dunster C., Kelly F.J., Poston L., Giussani D.A. (2010). Redox modulation of the fetal cardiovascular defense to hypoxemia. J. Physiol..

[37] Thakor A.S., Herrera E.A., Serón-Ferré M., Giussani D.A. (2010). Melatonin and vitamin C increase umbilical blood flow via nitric oxide-dependent mechanisms. J. Pineal. Res..

[38] Mahan D.C., Vallet J.L. (1997). Vitamin and mineral transfer during fetal development and the early postnatal period in pigs. J. Anim. Sci..

[39] Léger C.L., Dumontier C., Fouret G., Boulot P., Descomps B. (1998). A short-term supplementation of pregnant women before delivery does not improve significantly the vitamin E status of neonates--low efficiency of the vitamin E placental transfer. Int. J. Vitam. Nutr. Res..

[40] Herrera E., Ortega H., Alvino G., Giovannini N., Amusquivar E., Cetin I. (2004). Relationship between plasma fatty acid profile and antioxidant vitamins during normal pregnancy. Eur. J. Clin Nutr..

[41] Didenco S., Gillingham M.B., Go M.D., Leonard S.W., Traber M.G., McEvoy C.T. (2011). Increased vitamin E intake is associated with higher alpha-tocopherol concentration in the maternal circulation but higher alpha-carboxyethyl hydroxychroman concentration in the fetal circulation. Am. J. Clin. Nutr..

[42] Malone J.I. (1975). Vitamin passage across the placenta. Clin. Perinatol..

[43] Etzl R.P., Vrekoussis T., Kuhn C., Schulze S., Pöschl J.M., Makrigiannakis A., Jeschke U., Rotzoll D.E. (2012). Oxidative stress stimulates α-tocopherol transfer protein in human trophoblast tumor cells BeWo. J. Perinat. Med..

[44] Mohd Mutalip S.S., Ab-Rahim S., Rajikin M.H. (2018). Vitamin E as an antioxidant in female reproductive health. Antioxidants.

[45] Kasimanickam R.K., Kasimanickam V.R., Rodriguez J.S., Pelzer K.D., Sponenberg P.D., Thatcher C.D. (2010). Tocopherol induced angiogenesis in placental vascular network in late pregnant ewes. Reprod. Biol. Endocrinol..

[46] Kegley E.B., Ball J.J., Beck P.A. (2016). Impact of mineral and vitamin status on beef cattle immune function and health. J. Anim. Sci..

[47] Vieira-Filho L.D., Lara L.S., Silva P.A., Santos F.T.J., Luzardo R., Oliveira F.S.T., Paixão A.D.O., Vieyra A. (2011). Placental malnutrition changes the regulatory network of renal Na-ATPase in adult rat progeny: Reprogramming by maternal a-tocopherol during lactation. Arch. Biochem. Biophys..

[48] Chan A.C. (1993). Partners in defense, vitamin E and vitamin C. Can. J. Physiol. Pharmacol..

